# Biological and pharmacological roles of m^6^A modifications in cancer drug resistance

**DOI:** 10.1186/s12943-022-01680-z

**Published:** 2022-12-14

**Authors:** Zaoqu Liu, Haijiao Zou, Qin Dang, Hui Xu, Long Liu, Yuyuan Zhang, Jinxiang Lv, Huanyun Li, Zhaokai Zhou, Xinwei Han

**Affiliations:** 1 Department of Interventional Radiology, The First Affiliated Hospital of Zhengzhou University, Zhengzhou, 450052 Henan China; 2 Interventional Institute of Zhengzhou University, Zhengzhou, 450052 Henan China; 3 Interventional Treatment and Clinical Research Center of Henan Province, Zhengzhou, 450052 Henan China; 4 Center for Reproductive Medicine, The First Affiliated Hospital of Zhengzhou University, Zhengzhou, 450052 Henan China; 5 Department of Colorectal Surgery, The First Affiliated Hospital of Zhengzhou University, Zhengzhou, 450052 Henan China; 6 Department of Hepatobiliary and Pancreatic Surgery, The First Affiliated Hospital of Zhengzhou University, Zhengzhou, 450052 Henan China; 7 Department of Gastroenterology, The First Affiliated Hospital of Zhengzhou University, Zhengzhou, 450052 Henan China; 8 Department of Pediatric Urology, The First Affiliated Hospital of Zhengzhou University, Zhengzhou, 450052 Henan China

**Keywords:** Cancer drug resistance, m^6^A methylation, RNA modification, Chemotherapy, Immunotherapy

## Abstract

Cancer drug resistance represents the main obstacle in cancer treatment. Drug-resistant cancers exhibit complex molecular mechanisms to hit back therapy under pharmacological pressure. As a reversible epigenetic modification, N^6^-methyladenosine (m^6^A) RNA modification was regarded to be the most common epigenetic RNA modification. RNA methyltransferases (writers), demethylases (erasers), and m^6^A-binding proteins (readers) are frequently disordered in several tumors, thus regulating the expression of oncoproteins, enhancing tumorigenesis, cancer proliferation, development, and metastasis. The review elucidated the underlying role of m^6^A in therapy resistance. Alteration of the m^6^A modification affected drug efficacy by restructuring multidrug efflux transporters, drug-metabolizing enzymes, and anticancer drug targets. Furthermore, the variation resulted in resistance by regulating DNA damage repair, downstream adaptive response (apoptosis, autophagy, and oncogenic bypass signaling), cell stemness, tumor immune microenvironment, and exosomal non-coding RNA. It is highlighted that several small molecules targeting m^6^A regulators have shown significant potential for overcoming drug resistance in different cancer categories. Further inhibitors and activators of RNA m^6^A-modified proteins are expected to provide novel anticancer drugs, delivering the therapeutic potential for addressing the challenge of resistance in clinical resistance.

## Introduction

Estimated 600,000 people die from cancer each year, which is still a challenging problem that scientists are desperate to resolve [1, 2]. Oncotherapy is currently divided into five mainstream approaches: surgical resection, chemotherapy, radiotherapy, biological immunotherapy, and targeted therapy [3, 4]. Although there have been numerous breakthroughs for specific cancer categories, most strategies still are not as effective as expected. The major reason for treating cancer failure is the lacked understanding of the molecular mechanisms of therapeutic resistance. Resistance to chemotherapy drugs is usually divided into two main categories: acquired and intrinsic [5]. Intrinsic resistance, also called primary resistance, is a consequence of genetic alterations before treatment. Acquired drug resistance is caused by drug treatment and is also known as secondary resistance. Both are due to mutations and/or epigenetic changes in the genome of cancer cells. In the process of drugs binding to target and function, multiple mechanisms must be involved, including altered metabolism, transport, and varied target proteins [6]. Additionally, impaired apoptosis, augmented populations of cancer stem cells (CSCs), altered expression of oncogene/tumor suppressors, and manipulated tumor immune microenvironment (TIME) are also the dominant causes in charge of diminishing antitumor drug efficacy [7, 8]. Nevertheless, these are only influencing factors of therapy-resistant cancers, and the specific mechanism for therapy-resistant are unknown.

Researchers have identified more than 160 different chemically RNA modifications, creating a novel frontier called epitranscriptomics [9]. N^6^-methyladenosine (m^6^A) RNA modification has been identified as one of the most pervasive and abundant RNA modifications in eukaryotic messenger RNA (mRNA) [10, 11] and viral nuclear RNA [12, 13] since discovered in the 1970s. The process of m^6^A modification is dynamic and reversible, which is regulated by methylases (“writers”) and demethylases (“erasers”) (Table 1). m^6^A is installed by writers including methyltransferase-like (METTL) 3 [14], METTL14 [15], Wilms tumor 1-associated protein (WTAP) [17], KIAA1429 [18], METTL16 [16], RBM15 [20], and ZC3H13 [21]. m^6^A is removed by erasers such as fat mass and obesity-associated protein (FTO) [22] and alkB homolog 5 (ALKBH5) [23]. Different families of m^6^A reader proteins are capable of recognizing RNAs modified with m^6^A. One type of natural m^6^A reader protein contains the YT521-B homology (YTH) domain [33], and heterogeneous nuclear ribonucleoproteins (HNRNPs) belong to the other type, which mainly regulated alternative splicing or processing of target transcripts [29]. Other subfamily members are insulin-like growth factor 2 (IGF2) mRNA binding proteins (IGF2BP1/2/3) [31], and eIF3 [32].

**Table 1:** The role of m^6^A modification in the cancer biological functions

Type	m^6^A regulator	Activity	Ref
m^6^A writer	METTL3	catalyzes methylation reaction	[14]
	METTL14	assists METTL3 to recognize the subtract	[15]
	METTL16	catalyzes m^6^A modification	[16]
	WTAP	promotes METTL3-METTL14 heterodimer localization into nuclear speckles	[17]
	KIAA1429	directs the methyltransferase components to specific RNA region	[18]
	VIRMA	recruits the methyltransferase core components and associates with polyadenylation cleavage factors CPSF5 and CPSF6	[19]
	RBM15	binds the m^6^A complex and recruits it to a special RNA site	[20]
	ZC3H13	bridges WTAP to the mRNA-binding factor Nito	[21]
m^6^A eraser	FTO	reduces methylated bases	[22]
	ALKBH5	downregulates m^6^A modification level	[23]
m^6^A reader	YTHDC1	accelerates mRNA nuclear transport and alternative splicing	[24]
	YTHDC2	promotes the target RNA translation	[25]
	YTHDF1	enhances the translation of mRNA	[26]
	YTHDF2	increases mRNA degradation	[27]
	YTHDF3	mediates the translation or degradation	[28]
	HNRNPA2B1	promotes primary microRNA processing and mediates nuclear accumulation	[29]
	HNRNPC	mediates mRNA splicing and maturity	[30]
	IGF2BP1/2/3	enhances mRNA stability	[31]
	eIF3	enhances mRNA translation	[32]

**Fig. 1:**
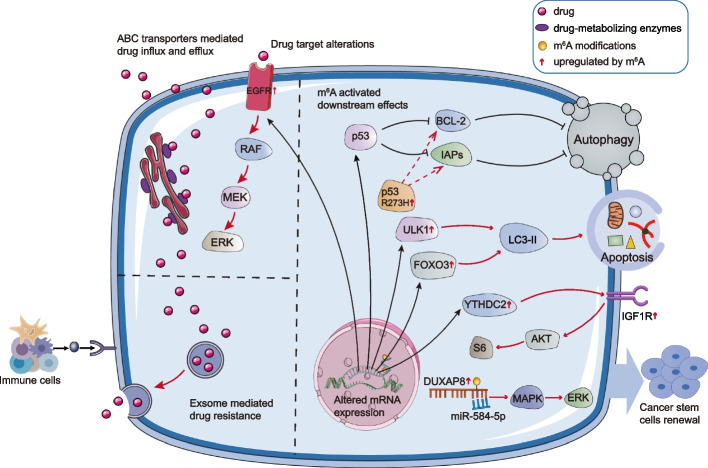
m^6^A-mediated biological processes of drug resistance. m^6^A was involved in several aspects of drug pharmacokinetics. m^6^A modifications upregulated drug transporters (e.g., ABCB1, ABCC1, ABCC10), facilitating ATP-driven drug efflux. m^6^A was also engaged in regulating several drug-metabolizing enzymes (e.g., CYP2C8 and UGT2B7) that affected the efficacy of chemotherapeutic drugs. Some drug targets (e.g., EGFR) were regulated by m^6^A and affected cancer development. Additionally, m^6^A also participated in activating downstream effects, which were embodied in the following three aspects. Firstly, m^6^A could selectively upregulate the p53 (R273 H) protein, releasing prohibited anti-apoptotic proteins (e.g., BCL-2, IAPs). Secondly, m^6^A altered the expression of various key signaling molecules (e.g., ULK1, FOXO3) in autophagy and ultimately regulated autophagy through light chain 3-II (LC3-II). Thirdly, m^6^A modification activated oncogenic bypass signaling through key molecules (e.g., IGF1R, DUXAP8) and promoted cell stemness, which became an important barrier to drug resistance. Immune cell infiltration and cytokine secretion in the tumor microenvironment were also regulated by m^6^A, which was relevant for cancer immunotherapy. The m^6^A modification of exosomal non-coding RNA was implicated in multiple biological processes in tumors and was associated with resistance to multiple anticancer drugs

Emerging evidence indicated that m^6^A modifications were strongly associated with therapy resistance. In several neoplasms, m^6^A regulators (writers, erasers, and readers) are frequently overexpressed, regulating oncoprotein expression, enhancing cancer inception, and cell multiplication [34]. m^6^A modulates multiple anticancer resistance, including drug transport and metabolism, target receptors, cancer stemness, DNA damage repair, and cell death [35–38]. In addition, m^6^A is closely related to the immune response in the tumor microenvironment, providing new prospects for tumor immunotherapy [39]. Importantly, small-molecule activators and inhibitors of m^6^A regulators have recently been revealed to possess considerable anticancer effects when applied alone or in combination with other anticancer agents, suggesting the novel function of m^6^A in anticancer drug resistance [40]. This review primarily introduced the significant role of m^6^A modification in tumor drug resistance, reviewed the mechanisms of RNA m^6^A modification associated with drug resistance, and further discussed the strategies targeting the m^6^A change in predicting and treating cancer resistance (Fig. 1).

## Mechanisms of m^6^A-mediated drug resistance

Cancer resistance is caused by a variety of factors, such as individual differences in drug sensitivity, tumor location, tissue spectrum, tumor aggressiveness, and alterations in intracellular molecules [3, 41]. The mechanism of m^6^A-mediated drug resistance was embodied in drug pharmacokinetics, tumor cells, and tumor microenvironment. Deciphering the impact of m^6^A modifications on the mechanisms of resistance to anticancer therapy could offer more prospects for individualized tumor treatment.

### m^6^A modulation in drug pharmacokinetics

#### m^6^A modulated aberrant drug transport and metabolism

Several membrane transporter proteins work together to promote drug efflux and resistance to chemotherapeutics. Most drug efflux experiments have focused on the role of the ATP-binding cassette (ABC) proteins [42]. Multidrug resistance (MDR) is mediated by a wide range of ABC transporters, such as ABCB1 (MDR1), ABCC1 (MRP1), ABCC10 (MRP7), and others [43, 44]. Recently, researchers have demonstrated that RNA m^6^A modifications regulated the expression of ABC family proteins through either direct impact on tumor transcripts or indirect effects on upstream signaling pathways. For instance, m^6^A upregulated estrogen-related receptor gamma (ERRγ) in chemo-resistant cancer cells. ERRγ not only directly enhanced ABCB1 transcription but also indirectly by further strengthening the interaction with p65 [45]. Besides, METTL3 m^6^A-dependently enhanced translation of ABCD1, leading to migration and spheroid formation in clear cell renal cell carcinoma (ccRCC) [46]. Notably, exosomal-FTO facilitated ABCC10 of recipient cells *via* FTO/YTHDF2/ABCC10 axis, eventually leading to gefitinib resistance in non–small cell lung cancer (NSCLC) [47]. Excluding drug transport, the efficacy of chemotherapeutic drugs is determined by the effects of drug metabolism, such as bioactivation, catabolism, conjugation, and elimination [48]. Recent studies have revealed that the m^6^A modification had a negative regulatory effect on regulating drug metabolism. For example, METTL3/14 depletion upregulated cytochrome P450 family member cytochrome P450 2C8 (CYP2C8), whereas FTO depletion suppressed it. Mechanically, YTHDC2 promoted *CYP2C8* mRNA degradation by recognizing the m^6^A in *CYP2C8* mRNA [49]. Another drug metabolism enzyme, carboxylesterase 2 (CES2), exhibits the exact mechanism of negative regulation by m6A as CYP2C8 [50]. UDP-glucuronosyltransferases (UGTs) are enzymes that catalyze the glucuronidation of various endogenous and exogenous compounds. In Huh-7 cells, the m^6^A regulator-mediated methylation modification also showed a negative correlation with UGT2B7 [51]. In summary, m^6^A modifications are novel regulators of drug transport and metabolism, contributing to the practice of personalized medicine.

#### m^6^A drove drug target alterations

Alterations to drug targets, such as mutations or changes in expression levels, impact drug response and resistance [52]. For example, the *TP53* gene coding for the p53 protein and mutant p53 proteins augmented cancer progression and generated drug resistance. METTL3-mediated m^6^A produced the p53 R273H mutant protein, causing MDR in colon cancer cells (Fig. 1) [53]. Epidermal growth factor receptor (EGFR) is another potential therapeutic target whose activation led to tumor cell proliferation, evasion of apoptosis, angiogenesis, and metastasis [54]. METTL3 augmented the translation efficiency of *EGFR*, followed by rebound activation of RAF/MEK/ERK, resulting in acquired PLX4032 resistance in melanoma (Fig. 1) [55]. Furthermore, YTHDF1 and YTHDF2 impacted cancer *via* binding m^6^A sites in the 3′-UTR of *EGFR* transcription and contributed to aberrant activities of downstream signal pathways [56, 57]. m^6^A-induced alterations in p53 protein and EGFR drug targets affect the efficacy of anticancer drugs, which may enable us to develop effective strategies to reverse the alterations in drug targets.

### m^6^A modulation in tumor cells

#### m^6^A regulated DNA damage repair

An ocean of chemotherapeutic agents primarily targeting genomic DNA can result in DNA lesions and inhibit transcription and replication [58]. m^6^A methyltransferase METTL3 facilitated oxaliplatin resistance in gastric cancer (GC) stem cells by substantial DNA damage repair [59]. Furthermore, METTL3 enhanced the expression of UBE2B, a crucial enzyme involved in DNA damage repair, thereby triggering multifarious drug resistance [60–62]. Additionally, other m^6^A regulators, YTHDF1 and ALKBH5, were also engaged in chemoresistance (including adriamycin, cisplatin, and olaparib) by enhancing DNA damage repair in breast cancer (BC) [63, 64].

#### m^6^A activated downstream effects

Anticancer drugs result in tumor cells’ death upon binding to their cellular targets. The m^6^A modification affected a diverse array of downstream impacts, including demolition of apoptosis, activation of autophagy, and energizing of oncogenic bypass signaling, which was a crucial part of current cancer therapy [65, 66].

##### m^6^A mediated cell apoptosis

Cell sensitivity to anticancer drugs was primarily determined by the upregulation of anti-apoptotic proteins, including B-cell lymphoma 2 (BCL-2), IAPs, and FLIP [67, 68]. Remarkably, m^6^A modification had a differential effect on BCL-2 expression according to the type of cancer. Recent research revealed that overexpression of FTO was accompanied by BCL-2 upregulation [69], which was consistent with the trend of regulation of BCL-2 by ALKBH5 found in epithelial ovarian cancer (EOC) [70]. Consequently, RNA m^6^A modification was inversely correlated with BCL-2 expression and anti-apoptosis. Nonetheless, varied results were found that m^6^A also positively influenced the expression of anti-apoptotic proteins. Wang et al. found METTL3 knockdown dramatically augmented apoptosis capabilities in BC by decreasing BCL-2 expression [71]. In esophageal cancer, NSCLC, and GC, reduced expression of m6A positively correlated with the decrease of the anti-apoptotic protein BCL-2, contributing to the activation of apoptosis [72–74]. Overall, the m^6^A modification modulated apoptosis based on the cancer context, uncovering the dual role of m^6^A in tumor cells.

##### m^6^A mediated cell autophagy

**Fig. 2:**
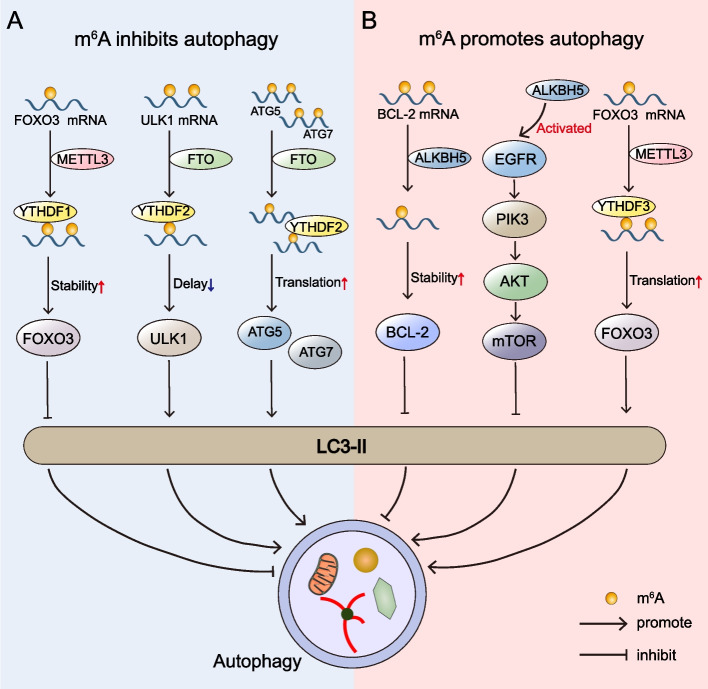
Dual effects of m^6^A in autophagy. On the one hand, the m^6^A modification inhibits autophagy. In hepatocellular carcinoma (HCC), METTL3 enhanced forkhead box O3 (*FOXO3*) mRNA stability and inhibited light chain 3-II (LC3-II) accumulation through a YTHDF1-dependent mechanism. The overexpression of FTO induced YTHDF2-dependent inhibition of unc-51-like kinase 1 (*ULK1*) mRNA decay and promoted LC3-II accumulation and autophagy. With the help of YTHDF2, FTO also increased the translation of autophagy-associated gene-5 (*ATG5*) and *ATG7* mRNAs and promoted autophagosome assembly. On the other hand, m^6^A modification also promotes autophagy. In epithelial ovarian cancer (EOC), ALKBH5 slowed autophagy by cementing B-cell lymphoma 2 (*BCL-2*) mRNA stability and activating the EGFR-PIK3CA-AKT-mTOR pathway. Additionally, the m^6^A reader YTHDF3 promoted autophagy through the upregulation of *FOXO3* mRNA translation

Autophagy is a lysogenic process that permits cells to own stress-coping strategies by degrading damaged organelles and accumulated proteins, which could result in cancer resistance treated with anticancer drugs [75–78]. m^6^A modification acted as a double-edged sword in autophagy regulation. In some cases, the RNA m^6^A modification inhibited autophagy (Fig. 2A). Light chain 3B (LC3B) was a well-known autophagy biomarker in the cytoplasmic matrix [79]. In hepatocellular carcinoma (HCC), METTL3 depletion promoted the LC3-II accumulation by reducing the stability of *FOXO3* mRNA through a YTHDF1-dependent mechanism [80]. Jin et al. [81] validated that FTO enhanced LC3B II accumulation by slowing the decay rate of unc-51-like kinase 1 (*ULK1*) transcripts in a YTHDF2-dependent manner. By the same mechanism, FTO enhanced the translation of autophagy-associated gene-5 (*ATG5*) and *ATG7* mRNAs and promoted an increase of LC3-II [82]. Conversely, m^6^A modification promoted autophagy in some cases (Fig. 2B). ALKBH5 activated the EGFR-PIK3CA-AKT-mTOR pathway and specifically cemented the *BCL-2* mRNA stability to slow the autophagy in EOC [70]. The latest study found that m^6^A reader YTHDF3 promotes autophagy by recognizing the METTL3-mediated m^6^A modification site around the *FOXO3* mRNA stop codon, providing new evidence for a dual role in m^6^A autophagy [83].

##### m^6^A regulated oncogenic bypass signaling

**Fig. 3:**
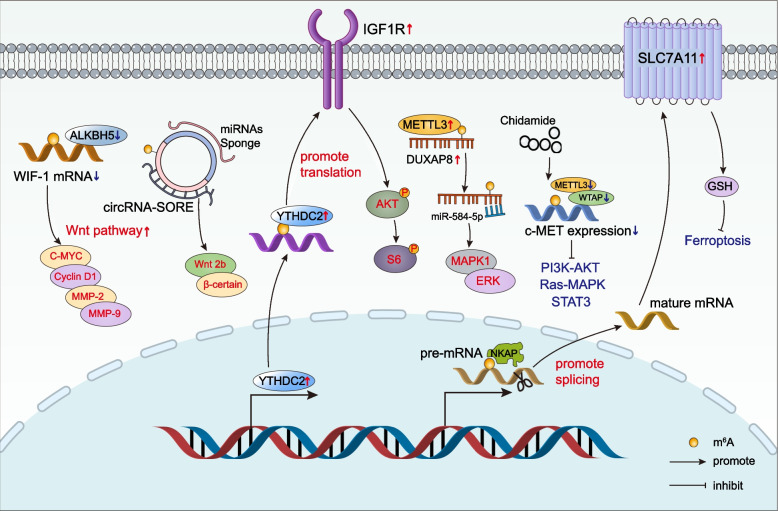
m^6^A-regulated oncogenic bypass signaling. Downregulation of ALKBH5 led to the downregulation of *WIF-1* mRNA expression, thus activating the Wnt pathway. The elevated levels of m^6^A in circRNA-SORE enhanced its stability and allowed it to competitively activate the Wnt/β-certain pathway by acting as a miRNA sponge. YTHDC2 promoted radiotherapy resistance by activating the IGF1R-AKT/S6 signaling axis. m^6^A modification-mediated DUXAP8 contributed to chemoresistance via miR-584-5p/MAPK1/ERK. Chidamide decreased c-MET expression and increased crizotinib sensitivity by reducing m^6^A methylation. NKAP promoted *SLC7A11* mRNA splicing and maturation, thereby inhibiting ferroptosis

Even though targeted therapies enabled tumor cells to be sensitive to chemotherapy, drug resistance remained a significant obstacle owing to the activation of oncogenic bypass pathways (including Wnt/β-catenin, PI3K/AKT, MAPK, or c-MET signaling) [84–86]. ALKBH5 suppressed m^6^A modification of the *WIF-1* mRNA to promote its transcription, which probably interfered with the Wnt signaling and led to chemosensitivity [87]. Besides, Xu et al. [88] revealed that the elevated level of m^6^A in circular RNA (circRNA)-SORE enhanced its stability, allowing it to induce sorafenib resistance by acting as a microRNA (miRNA) sponge to isolate miR-103a-2-5p and miR-660-3p, thereby competitively activating the Wnt/β-catenin pathway. YTHDC2, the m^6^A reader protein, regulated irradiation efficacy *via* IGF1R-AKT/S6 pathway, leading to radiotherapy resistance of nasopharyngeal carcinoma (Fig. 1) [89]. Alternatively, m^6^A modification-mediated *DUXAP8* regulated malignant phenotype and chemoresistance of HCC through miR-584-5p/MAPK1/ERK pathway (Fig. 1) [90]. Beyond that, chidamide reduced c-MET expression by lowering m^6^A methylation, which increased crizotinib sensitivity in NSCLC cells in a c-MET/HGF-dependent manner [91]. NF-κB activating protein (NKAP), as a reader of m^6^A, promoted *SLC7A11* mRNA splicing and maturation, thereby enhancing cell resistance to ferroptosis inducers [92]. Overall, the m^6^A mutation activated the oncogenic bypass pathway, circumventing the classical drug targets, which could be considered in targeted therapy to avoid or overcome drug resistance (Fig. 3).

#### m^6^A affected the sustainment of cell stemness

CSCs represent a small population of tumor cells sustaining versatility and promoting tumor progression and drug resistance [93, 94]. METTL3 was involved in regulating the stemness and chemosensitivity of colon cancer through the upregulation of LGR5 [95]. Aside from that, METTL3 facilitated oxaliplatin resistance in CD133+ stem cells by promoting PARP1 mRNA stability and increased base resection repair pathway activity [59]. Liu and his team [96] identified a crucial regulatory METTL14-miR99a-5p-TRIB2 feedback circuit that promoted cancer stemness and radioresistance in esophageal squamous cell carcinoma (ESCC). m^6^A modification of circHPS5 expedited cytoplasmic output and facilitated (epithelial-to-mesenchymal transition) EMT and CSC phenotypes, further accelerating HCC cell tumorigenesis [97]. HNRNPA2B1 promoted CD44^+^/CD24^−^/^low^ CSC and altered the EMT markers to initiate acquired endocrine resistance by activating ser/thr kinase growth factor signaling pathways [98]. The researches about m^6^A and stemness are still quite insufficient; thus, linking m^6^A modifications to CSCs in tumor drug resistance may be a new direction for future studies.

### m^6^A modulation in the tumor microenvironment

#### m^6^A altered the TIME

**Fig. 4:**
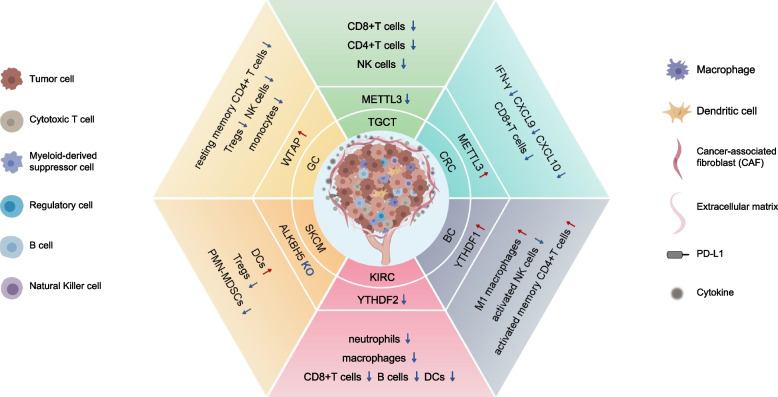
m^6^A-mediated alterations in the tumor immune microenvironment. METTL3 was significantly downregulated in testicular germ cell tumor tissues, which positively correlated with the level of tumor infiltration by CD8+ T cells, CD4+ T cells, and natural killer (NK) cells. WTAP was overexpressed in granulosa cells (GCs) and negatively correlated with T cell infiltration and T cell-induced immunity. In skin cutaneous melanoma (SKCM) patients, the number of infiltrating regulatory T cells (Tregs) and polymorphonuclear myeloid-derived suppressor cells (PMN-MDSCs) was significantly decreased in ALKBH5 knockout (KO) tumors, while dendritic cells (DCs) were significantly elevated. In kidney renal clear cell carcinoma (KIRC), downregulation of YTHDF2 positively correlated with lymphocyte infiltration (e.g., B cells, T cells, macrophages, neutrophils, and dendritic cells). In breast cancer (BC), high expression of YTHDF1 distinctly exhibited higher infiltration scores of activated memory CD4 + T cells and M1 macrophages but low infiltration levels of activated NK cells. METTL3 was highly expressed in mismatch-repair-proficient or microsatellite instability-low colorectal cancer (CRC) patients, and decreased interferon-γ (IFN-γ) Chemokine (C-X-C motif) ligand 9 (CXCL9) and CXCL10 secretion in TIME

An increasing number of studies demonstrated that the alteration of m^6^A regulated the TIME features [99], making the m^6^A regulator a promising immunotherapy target. Abnormal expression of METTL3 in various cancers played a dual part in the infiltration of immune cells. On the one hand, METTL3 was significantly downregulated in testicular germ cell tumor tissues, which positively correlated with the tumor-infiltrating levels of CD8+ T cells, CD4+ T cells, and NK cells [100]. On the other hand, the depletion of METTL3 or METTL14 tumors increased the infiltration of cytotoxic CD8+ T cells and elevated secretion of interferon-gamma (IFN-γ), CXCL9, and CXCL10 in the TIME, thus enhancing the reaction to anti-programmed cell death protein 1 (PD-1) treatment in pMMR-MSI-L colorectal cancer (CRC) [101]. WTAP was overexpressed in GC and negatively associated with T cell infiltration and T cell-induced immunity, indicating an unfavorable prognosis [102]. The depletion of FTO reprogrammed the immune response and enhanced T-cell toxicity by suppressing the expression of immune checkpoint genes, especially *LILRB4* [103]. In melanoma, combining FTO inhibition with blocking the PD-1/PD-L1 checkpoint may relieve the resistance to immunotherapy [104]. In addition to regulating immune checkpoint blockade, FTO functioned as an essential epitranscriptomic regulator by regulating glycolytic metabolism and suppressing the function of CD8+ T cells [105]. ALKBH5, another m^6^A eraser, correlated positively with Treg cell infiltration. Melanoma patients treated with anti-PD-1 therapy benefited from ALKBH5 deletion [106]. Furthermore, the latest research found that a large number of immune checkpoint receptors (including PD-1, TIM-3, and CTLA-4) as well as lymphocytes infiltrating (such as B cells, T cells, macrophages, and dendritic cells) positively correlated with the level of m^6^A readers YTHDF1, and YTHDF2 in respective cancer type, including glioma, NSCLC, kidney renal clean cell carcinoma and BC [107–110]. Despite the different TIME among tumor types and individual responses, correcting m^6^A regulator disorder was a feasible strategy for cancer immunotherapy (Fig. 4).

#### m^6^A modified exosomal non-coding RNA

Exosomes are nano-sized extracellular vesicles that contain constituents of origin cells, which are essential for tumor-stroma cellular communication for mediating pigmentation-induced tumor resistance [111, 112]. However, the role of exosomal non-coding RNAs on tumor drug resistance has not been investigated until recently. Liu and colleagues [113] identified METTL3 positively modulated pri-miR-320b maturation process, which was associated with peritumoral lymphangiogenic activity and lymph node metastasis. Besides, METTL3 promoted the exosomal miR-181b-5p in cancer-associated fibroblasts (CAFs) and suppressed CRC cell sensitivity to 5-fluorouracil (5-FU) *via* the METTL3/miR-181d-5p axis [114]. In NSCLC, the miR-4443 level was significantly upregulated in cisplatin-resistant tumor-released exosomes. Mechanistically, overexpression of miR-4443 inhibited FSP1-mediated ferroptosis induced by cisplatin treatment *in vitro* and promoted tumor growth *via* METLL3-mediated m^6^A manner *in vivo* [115]. Exosome-transmitted circVMP1 was also involved in cisplatin resistance by targeting the miR-524-5p-METTL3/SOX2 axis [116]. Another research showed that exosomal long-noncoding RNAs (lncRNAs) might be a controller in regulating drug resistance. They discovered adipocyte exosomes contained the LncRNA package released by multiple myeloma (MM) cells through METTL7A-mediated methylation resulting in therapeutic resistance [117].

### m^6^A induced specific drug resistance

**Fig. 5:**
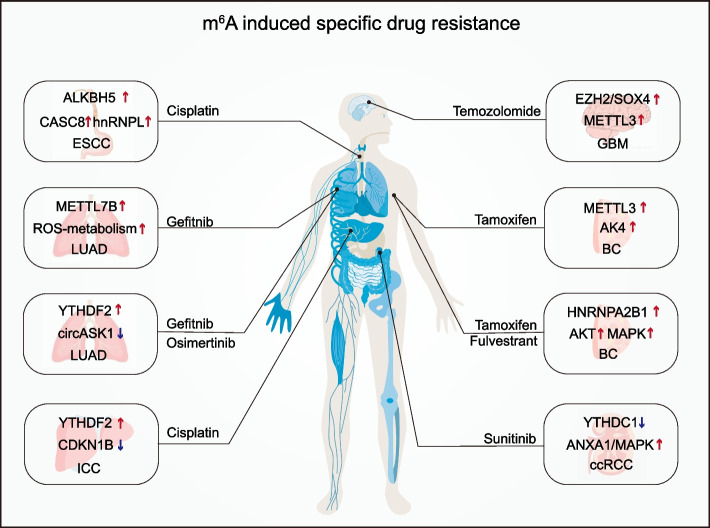
m^6^A-induced specific drug resistance. Specific chemotherapy drug resistance associated with m^6^A and related regulators in esophageal squamous cell carcinoma (ESCC), lung adenocarcinoma (LUAD), intrahepatic cholangiocarcinoma (ICC), glioblastoma multiforme (GBM), breast cancer (BC), clear cell renal cell carcinoma (ccRCC)

Emerging researches show that m^6^A RNA methylation is involved in drug resistance of multiple cancer chemotherapeutic agents by regulating the expression of different targets or pathways. Elevated levels of m^6^A due to METTL7B overexpression in lung adenocarcinoma (LUAD) induced gefitinib and osimertinib resistance in a ROS-scavenging-dependent manner [118]. YTHDF2-mediated endoribonucleolytic cleavage of m^6^A-modified circASK1 also contributed to LUAD gefitinib resistance [119]. ALKBH5-mediated m^6^A demethylation stabilizes *CASC8* transcription, ultimately leading to cisplatin resistance in ESCC [120]. Furthermore, YTHDF2 increased *CDKN1B* mRNA degradation in an m^6^A-dependent manner, which promoted intrahepatic cholangiocarcinoma (ICC) progression and reduced sensitivity to cisplatin treatment [121]. m^6^A modifications also play an integral part in tamoxifen resistance, a classical chemotherapeutic agent in breast cancer treatment [122]. METTL3 promoted the translation of *AK4* mRNA by increasing m^6^A levels and facilitated ROS production and activation of p38, ultimately resulting in tamoxifen resistance [123]. Tamoxifen resistance was also caused by the m^6^A reader HNRNPA2B1 regulating downstream targets through activation of the ser/thr kinase growth factor signaling pathway [98]. In treating glioblastoma multiforme (GBM) with temozolomide, METTL3 increased the m6A modification of histone modify-related gene transcripts leading to the development of chemoresistance [124]. In ccRCC, YTHDC1 acted as an m^6^A reader and regulated the sensitivity of tyrosine kinase inhibitors (TKI) such as sunitinib through the YTHDC1/ANXA1 axis [125]. In conclusion, research on the molecular mechanisms of m^6^A in different chemotherapeutic agents has attracted increasing attention, offering new prospects and potential therapeutic targets for reversing therapeutic resistance (Fig. 5).

## Targeting the m^6^A modification to surmount anticancer resistance

As discussed above, m^6^A modifications had a dual function in driving drug resistance, yet obscure behind the molecular mechanisms. In addition to mutations in m^6^A, each tumor’s m^6^A regulators had a different function [126], drawing researchers’ attention to the regulating agency of m^6^A regulators in targeted therapy (Table 2).

**Table 2:** The role and regulatory mechanism of m^6^A regulator in cancer drug resistance

m^6^A regulator	Cancer type	Role in cancer	Expression in cancer drug resistance	Drug	Target genes	Mechanism	Ref
METTL3	BC	Oncogene	High	Adriamycin	MALAT1	METTL3 promoted MALAT1 protein and activated MALAT1/E2F1/AGR2 axis	[127]
METTL3	NSCLC	Oncogene	NA	Cisplatin	YAP	METTL3 enhanced the translation of YAP mRNA by recruiting YTHDF1/3 and eIF3b	[128]
METTL3	HCC	Oncogene	Low	Sorafenib	FOXO3	METTL3 promoted FOXO3 stability through a YTHDF1-dependent mechanism	[80]
METTL3	HCC	Oncogene	High	Adriamycin	ERRγ	METTL3 delayed the half-life of precursor mRNA of ERRγ	[45]
METTL3	CRC	Oncogene	NA	Oxaliplatin or irinotecan	CBX8	METTL3 enhanced CBX8 mRNA stability through an IGF2BP1-dependent mechanism	[95]
METTL3/14	CRC	NA	NA	anti-PD-1 antibodies	STAT1 and IRF1	METTL3 or METTL14 loss promoted IFN-c-Stat1-Irf1 signaling through stabilizing the Star1 and Irf1 mRNA *via* YTHDF2	[101]
METTL3,WTAP	NSCLC	Oncogene	NA	Crizotinib	c-MET	The downregulation of METTL3 and WTAP decreased c-MET expression	[91]
WTAP	BLCA	Oncogene	High	Cisplatin	TNFAIP3	Circ0008399 bound to WTAP and activated the circ0008399/WTAP/TNFAIP3 pathway	[129]
WTAP	NKTCL	Oncogene	High	Cisplatin	DUSP6	WTAP enhanced DUSP6 expression	[130]
WTAP	BC	Oncogene	High	Adriamycin	DLGAP1-AS1	WTAP motivated DLGAP1-AS1 stability	[131]
FTO	GBM	Oncogene	NA	Temozolomide	PDK1	JPX interacted with FTO and degraded PDK1 expression	[132]
FTO	MM	Oncogene	High	Bortezomib	SOD2	FTO downregulated the expression of SOD2	[133]
FTO	BC	Oncogene	High	Doxorubicin	STAT3	FTO could activate STAT3 signaling in BC cells	[134]
FTO	CSCC	Oncogene	High	Cisplatin	β-Catenin	FTO promoted gene expression of β-catenin *via* m^6^A modification	[135]
FTO	Leukemia	Oncogene	High	Imatinib, nilotinib, or PKC412	MERTK and BCL-2	m^6^A demethylated by FTO promoted MERTK and BCL-2 stability	[69]
ALKBH5	PC	Tumor suppressor	Low	Gemcitabine	WIF-1	ALKBH5 promoted WIF-1 transcription to hinder Wnt signaling	[87]
ALKBH5	EOC	Oncogene	High	Cisplatin	JAK2	The ALKBH5-HOXA10 loop jointly activated the JAK2/STAT3 signaling pathway	[136]
ALKBH5	T-ALL	Oncogene	High	Glucocorticoid	USP1	ALKBH5 increased USP1 and Aurora B expression	[137]
ALKBH5	EOC	Oncogene	Low	Olaparib	FZD10	Downregulation of FTO and ALKBH5 contributed to FZD10 mRNA upregulation	[138]
ALKBH5	OSCC	Oncogene	High	Cisplatin	FOXM1	ALKBH5 promoted FOXM1 expression by demethylating its nascent transcripts	[139]
YTHDF1	NSCLC	Oncogene	Low	Cisplatin	Keap1	YTHDF1 promoted the translational efficiency of Keap1	[140]
IGF2BP3	CRC	NA	High	Doxorubicin	ABCB1	IGF2BP3 promoted the stability and expression of ABCB1 mRNA	[141]
HNRNPC	GC	Oncogene	High	5-FU, paclitaxel, or cisplatin	NA	mAb 5B2 targeted HNRNPC overexpressed in chemo-resistant GC cells	[30]

*NA* Not reported

### Targeting methyltransferase

#### METTL3

As an m^6^A writer, METTL3 regulated cancer initiation and progression, including glioblastoma, BC, HCC, leukemia, and other cancer cells [142–145]. Silencing METTL3 could reverse cancer cells’ resistance to radiotherapy/chemotherapy even though its biological effects were likely organ/lineage-specific. A recent study proposed that the elevated expression of METTL3 enhanced *SOX2* mRNA stability. Specifically, silencing METTL3 enhanced the sensitivity of (glioblastoma stem cells) GSCs to γ-H2AX and efficient DNA repair, resulting in rescuing glioblastomas’ radiosensitivity [146]. Furthermore, silencing METTL3 promoted temozolomide’s sensitivity, inhibited proliferation, and facilitated apoptosis. Taketo’s study [62] showed that cancer cells were more sensitive to chemotherapy and radiotherapy when METTL3 was suppressed. Their study affirmed that METTL3 was linked to the alternative expression of MAPK cascades, especially in patients treated with gemcitabine, 5-FU, and cisplatin. Meanwhile, Uddin and colleagues [53] demonstrated that METTL3 catalyzed a preferential pre-mRNA splicing in the point-mutated codon 273 (G > A) of *TP53*. Whereafter, the enlarged translation of mutant p53 protein-induced MDR as a result. m^6^A was recruited to the translation initiation complex in a METTL3-mediated manner and directly promoted yes-associated protein (*YAP*) translation. Additionally, the stability of MALAT1 was increased by METTL3/YTHDF3 complex, which also promoted YAP expression *via* the MALAT1-miR-1914-3p-YAP axis. The amplified YAP expression induced DDP resistance and metastasis [128]. Meanwhile, m^6^A also developed resistance to other chemotherapeutic drugs in NSCLC. Chidamide downregulated c-MET expression by decreasing its mRNA m^6^A methylation, thereby increasing the sensitivity of NSCLC cells to crizotinib in a c-MET−/HGF-dependent manner [91]. By eliminating METTL3-mediated *FOXO3* mRNA stabilization in the hypoxic tumor microenvironment, METTL3 depletion significantly enhanced the drug resistance of HCC to sorafenib, which confirmed FOXO3 as a crucial m^6^A modification downstream molecule in the sorafenib resistance of HCC [80]. The latest study revealed the potential function of METTL3 in adriamycin resistance (ADR) in BC. METTL3-mediated m^6^A regulated MALAT1 expression, thereby recruiting E2F1 and promoting AGR2 expression, which resulted in ADR in BC [127]. A recent study in GC showed that the reader IGF3BP1 recognized METTL3-mediated m6A modification on apoptotic protease-activating factor 1-binding lncRNA to maintain its stability, which inhibited GC cell apoptosis and led to multidrug resistance [147]. Notably, m^6^A-targeted transcription factors differed across cancer phenotypes, and further studies on the regulatory mechanism of action are necessary to develop more treatments targeting METTL3.

#### WTAP

WTAP is another essential m^6^A methyltransferase complex interacting with METTL3 and METTL14 to pre-RNAs/hnRNAs for catalytic activity. The targeting WTAP knockdown significantly reduced m^6^A modification and increased apoptosis [17]. Bansal et al. [148] hypothesized that excessive expression of the WTAP was associated with an oncogenic role in leukemogenesis. Its abnormal elevated expression correlated with a poor prognosis of acute myeloid leukemia (AML). They also predicted that WTAP was an HSP90 client protein, which maintained the stability of many oncoproteins and inhibited the anticancer efficiency of etoposide. After silencing WTAP, K562 cells showed significant apoptosis activity after etoposide treatment. A combined application of etoposide and WTAP inhibitors would escalate AML cell apoptosis. Circ0008399 (a novel circular RNA) promoted the expression of the target gene TNFAIP3 by increasing its mRNA stability in an m^6^A-dependent manner. As a result, WTAP diminished bladder cancer (BLCA) chemosensitivity to CDDP *via* the circ0008399/WTAP/TNFAIP3 pathway [129]. Ma et al. [130] suggested that WTAP-mediated DUSP6 upregulation contributed to carcinogenesis and drug resistance of nasal-type natural killer/T-cell lymphoma, providing a rationale for developing innovative avenues of antitumor therapeutics for natural killer/T-cell lymphoma (NKTCL). Likewise, WTAP bound to the m^6^A modified site of DLGAP1-AS1 contributed to stability, promoting BC-ADR through WTAP/DLGAP1-AS1/miR-299-3p feedback loop [131].

### Targeting demethylase

#### FTO

Demethylase FTO played an oncogenic role in BC, AML, and other malignant tumors [149–151]. FTO-mediated m^6^A modification was also associated with drug resistance in various cancers, such as MM, glioblastoma, and melanoma. YAN et al. [69] confirmed that the TKI-tolerance phenotype emerged in leukemia patients because the overexpression of FTO caused m^6^A reduction. Signal transducers and activators of transcription 3 (STAT3) were constitutively active in several cancer types, and such hyperactivity was associated with an adverse clinical outcome [152]. Wang et al. [134] found increased expression of FTO and STAT3 in doxorubicin-resistant BC cells, and STAT3 bound to the FTO promoter to positively accommodate FTO expression. Moreover, FTO was involved in STAT3-mediated doxorubicin resistance and impaired doxorubicin sensitivity in BC cells. The overexpressing of FTO in cervical squamous cell carcinoma (CSCC) was resistant to radiotherapy and chemotherapy by the FTO-mediated mRNA demethylation and ERCC1 activity [135]. Interestingly, FTO was set up at high concentrations in patients’ MM cells and bone marrow tissues. Further analysis showed that FTO promoted bortezomib resistance by destabilizing SOD2 expression through an m^6^A-dependent manner, which might open up innovative therapeutic options [133]. JPX, a non-coding RNA adjacent to the X-inactive specific transcript, was entangled in tumor progression. It appeared that JPX interacted with the mRNA of phosphoinositide-dependent kinase-1 (*PDK1*) and promoted its stability and expression. Furthermore, JPX demethylated *PDK1* mRNA, through its interaction with FTO alpha-ketoglutarate-dependent dioxygenase, contributed to the enhanced demethylation. Consequently, JPX exerted its GBM positive effects *via* the FTO/PDK1 axis and directly stabilized the *PDK1* mRNA in temozolomide drug resistance [132]. Besides, the knockdown of FTO decreased the stability of PD-1, CXCR4, and SOX10, increasing RNA attenuation *via* m^6^A reader YTHDF2. It also sensitized melanoma cells to IFN-γ and anti-PD-1 therapy.

#### ALKBH5

ALKBH5, another m^6^A modification demethylase, was related to the onset, development, and prognosis of colon cancer, BLCA, EOC, and oral squamous cell carcinoma (OSCC) [153–155]. The downregulation of FTO and ALKBH5 in ovarian cancers with breast-cancer susceptibility gene 2 (*BRCA2*) mutations enhanced *FZD10* mRNA m^6^A modifications, which ultimately reduced the sensitivity of PARPi *via* the Wnt/β-catenin pathway [138]. Moreover, ALKBH5 promoted cisplatin resistance in cancer cells [136]. HOXA10, the upstream transcription factor of ALKBH5, could form a loop with ALKBH5. In this way, ALKBH5 and HOXA10 together activated the JAK2/STAT3 signaling pathway, mediating *JAK2* m^6^A demethylation and promoting EOC resistance to cisplatin. A recent study found that ubiquitin-specific proteases (USPs) were associated with T-cell acute lymphoblastic leukemia (T-ALL) occurrence and chemoresistance. ALKBH5 exhibited a carcinogenic effect on cancers and improved *USP* mRNA’s stability, resulting in GC resistance [137]. Multiple neoplasms expressed the human RNA helicase DDX3, essential for cell proliferation, invasion, and metastasis. By directly regulating ALKBH5, DDX3 could decrease m^6^A methylation of *FOXM1* and *NANOG* transcripts, giving rise to cisplatin resistance in OSCC cells [139]. Likewise, the deletion of the m^6^A demethylase ALKBH5 sensitized tumors to cancer immunotherapy, suggesting that ALKBH5 may be a potential target to improve the outcome of immunotherapy for melanomas, CRC, and other underlying cancers [106]. In pancreatic cancer (PC), ALKBH5-mediated m6A modification caused DDIT4-AS1 overexpression, and DDIT-AS1 increased cancer stemness and led to gemcitabine resistance by destabilizing DDIT4 and activating the mTOR pathway [156].

### Targeting other m^6^A regulators

So far, strategies targeting m^6^A mainly relied on the regulation of methyltransferase (such as METTL3 and WTAP) and demethylase. However, multiple sources of evidence suggested that other m^6^A modulators also had great potential as drug-therapeutic targets. For instance, the depletion of METTL14, core subunits of RNA methyltransferase, dramatically slowed tumor growth and prolonged the survival in mice bearing CT26 CRC and B16 melanoma [101]. m^6^A reader protein also played a pivotal role in drug resistance. In NSCLC, Keap1 was degraded following YTHDF1 depletion, facilitating Keap1-Nrf2-AKR1C1 axis cells and resulting in cisplatin resistance [140]. MicroRNA-145 could abrogate YTHDF2’s role as an oncogene in HepG2 cells associated with HCC [157]. In CRC, hypoxia-induced antisense lncRNA STEAP3-AS1 competed with YTHDF2 to *STEAP3* mRNA binding site, protecting *STEAP3* mRNA from m^6^A-mediated degradation and leading to high STEAP3 protein expression. Followed by this, activation of the Wnt/β-catenin pathway contributed to CRC progression [158]. Moreover, paclitaxel, 5-FU, and cisplatin were more effective in cell lines that lacked the m^6^A reader protein HNRNPC [30]. IGF2BP3, another m^6^A reader, was bound to the m^6^A modification region of *ABCB1* mRNA and increased chemoresistance in CRC cells [141]. These studies illustrated that HNRNPC and IGF2BP3 could be latent biomarkers for chemoresistance.

### m^6^A-targeted compounds

#### FTO inhibitors

Rhein was the first identified inhibitor for FTO *in vitro* and *in vivo*, which was neither a structural mimic of 2OG nor a chelator of the metal ion. Rhein blocked FTO demethylase by competitively binding its catalytic domain instead [159]. In therapy, the rhein-TKI combination synthetically eradicated relapsed/refractory leukemia [69], while rhein exposure increased the level of m^6^A in leukemia. In contrast, no growth arrest was observed after 24 hours of 20 μM rhein, proposing the anticancer therapy of rhein. Ascorbic acid also enhanced the activity of 2OG-dependent dioxygenases. In BC, ascorbic acid analog MO-I-500 exhibited antiproliferative activity in an FTO-dependent manner [160, 161]. However, rhein, as well as MO-I-500, was a broad-spectrum 2-OG inhibitor, which tremendously reduced their applications. In a high-throughput fluorescence polarization assay, meclofenamic acid (MA), a non-steroidal anti-inflammatory drug, was selected as the inhibitor of FTO. Moreover, the ethyl ester form of MA (MA2) upgraded levels of m^6^A modification in mRNA [162]. Additionally, MA2 inhibited self-renewal and tumorigenesis of GSCs in a GSC-xenograft mouse model and prolonged survival [163]. Of note, MA2 enhanced the antitumor effect of chemotherapy in glioma [164]. As a result of the specific inhibitory property of MA, higher potency derivatives were designed and synthesized. A new MA-derived inhibitor, FB23, directly bound to FTO and selectively inhibited its activity, which possessed 140-fold over that of MA. The benzohy-droxamic acid, termed FB23–2, was a further practical analog of FB23 [165]. FB23–2 exhibited FTO-dependent anti-leukemia effects broadly and targeted the same signaling pathways as FB23. Dac51, another small-molecule analog of FB23, could modulate the tumor microenvironment *via* inhibiting FTO and mounting CD8+ T cell infiltration, contributing to a remarkable antitumor efficac y[105]. FTO-04 demonstrated robust inhibition of neurosphere formation in patient-derived GSCs but did not inhibit the growth of healthy human neural stem cells. On the side, FTO-04-mediated inhibition of FTO increased m^6^A modification and demethylated N6,2′-O-dimethyladenosine (m^6^A_m_) levels of GSCs [166]. Nafamostat mesylate often was applied in treating pancreatitis and cancers. The combination of thermodynamic and enzymatic activity provided insight into the FTO inhibition of nafamostat mesylate [167]. R-2-hydroxyglutarate (R-2HG) was architecturally and chemically similar to another inhibitor, 2OG. R-2HG inhibited FTO’s enzymatic activity by competitive inhibition and proved the overall antitumor effect. As a result of the R-2HG therapeutic regimen, m^6^A modification levels increased. Meanwhile, aerobic glycolysis was suppressed by inhibiting FTO activity and downstream signaling molecules, consisting of MYC, CEBPA, PFKP, and LDHB [168, 169]. CS1 and CS2 displayed a much higher efficacy. Consequently, two highly efficacious FTO inhibitors were named CS1 and CS2. They displayed a much higher efficacy in inhibiting AML cells’ viability than two previously reported FTO inhibitors (FB23–2 and MO-I-500) [103]. Therefore, FTO represented a modern therapeutic potential to target cancer therapy, and more clinical studies were required to confirm the long-term side effects of these inhibitors.

#### METTL3 inhibitors

Bedi et al. [170] reported a virtual screening method for almost 4000 adenosine derivatives to identify potential METTL3 inhibitors. Their best compound, S-adenosyl-L-methionine (SAM) mimic, was the first small molecule to inhibit METTL3. METTL3 inhibitors possessed excellent ligand efficiency, and their binding patterns were validated by protein crystallography. Respective RNA m^6^A methyltransferase inhibitors displayed anticancer abilities. Accompanied by the selective reduction of m^6^A levels on known leukemogenic mRNAs, STM2457 treatment reduced AML growth and increased differentiation and apoptosis [171]. Another METTL3 chemical inhibition, UZH1a, reduced the m^6^A/A ratio in mRNAs of different cell lines, revealing the potential implications of METTL3 inhibition in tremendous disease models [172].

#### Other m^6^A regulator activators and inhibitors

Using silico-based discovery could identify small-molecule ligands binding to the METTL3–14-WTAP complex. Primarily, SAM bonded with Asp377 and acted as a hydrogen bond donor to the Asp395 of METTL3 protein. Similarly, four compounds bound to the extent of the METTL3 enzyme relating to Asp295, Phe534, Arg536, and Asn539. METTL3-METTL14 RNA m^6^A methyltransferase complex activators provoked cells to modify mRNA m^6^A [173]. Their potential anticancer effects needed more experiments to prove. Li and his team [106] identified a small molecule inhibitor of ALKBH5 by using the X-ray crystal structure in silico screening of compounds and named ALK-04. Compound libraries verified this specific inhibitor. Subsequent proof found that melanoma tumor growth was significantly reduced in mice applying the ALK-04 compared to the control group. This study also provided evidence for ALKBH5 inhibitors combined with immunotherapy against melanoma. BTYNB has been identified by compound library screening with its ability to inhibit c-Myc and IGF2BP1 protein selectively [174]. The small molecule BTYNB also destabilized *E2F1* mRNAs by impairing the IGF2BP1-RNA association, which interfered with cellular protein synthesis and tumor growth [174]. Table 3 collates the identified m^6^A-targeted compounds.

**Table 3:** Identified m^6^A-targeted compounds

Molecule	Target	Activity	IC_50_ (of target) (μM)	Mechanism in cancer/ cell line	Validated cancer type/ cell line type	Identified year	Ref
rhein	FTO, ALKBH2, ALKBH3	inhibit	21 (FTO)	rhein restored nilotinib resistance by inhibiting the activity of FTO	leukemia	2012	[69, 159]
MO-I-500	FTO	inhibit	8.7	MO-I-500 inhibited BC cells survival and colony-forming	BC	2014	[160, 161]
MA2	FTO	inhibit	7	MA2 treatment inhibited GSCs growth and self-renewal	GBM	2014	[162, 166]
FB23–2	FTO	inhibit	0.06	FB23–2 suppressed proliferation and promoted the differentiation and apoptosis of AML cells	AML	2019	[165]
Dac51	FTO	inhibit	0.4	Dac51 increased CD8 + T cell infiltration and synergized with anti-PD-L1 blockade	SKCM, lung cancer	2021	[105]
FTO-04	FTO, ALKBH5	inhibit	3.39 (FTO)	prevented neurosphere formation in patient-derived GSCs	GBM	2021	[166]
R-2HG	FTO	inhibit	133.3	R-2HG inhibited cancer cells proliferation/survival by targeting FTO/m^6^A/MYC/CEBPA pathway	AML	2018	[168]
CS1	FTO FTO	inhibit	0.143	CS1 and CS2 exerted anti-leukemic effects by activating apoptosis signaling and inhibition of MYC pathways	AML AML	2020	[103]
CS2		inhibit	0.713			2020	[103]
adenosine	METTL3	inhibit	500	NA	NA	2020	[170]
STM2457	METTL3	inhibit	0.0169	STM2457 reduced AML growth and increased differentiation and apoptosis	AML	2021	[171]
U2H1a	METTL3	inhibit	7	U2H1a reduced m^6^A/A levels in mRNA fraction	AML, osteosarcoma, HEK293T	2021	[172]
ALK-04	ALKBH5	inhibit	NA	ALK-04 reduced tumor growth and enhanced the efficacy of anti–PD-1 therapy	melanoma, CRC	2019	[106]
BTYNB	IGF2BP1	inhibit	6	BTYNB impaired tumor cell proliferation and inhibited E2F-driven gene expression	HepG2, A549, ES-2, PANC-1, MV3	2017	[175]
METTL3/14-WTAP compounds	METTL3	activate	0.281	The compounds increased the mRNA m^6^A levels and regulated the cell cycle	HEK293 cell	2019	[173]
MPCH	METTL3/14	activate	NA	MPCH activated METTL3/14 and resulted in considerable m^6^A hypermethylation after short UV light exposure	A549, MCF-7, HeLa	2021	[176]
IDH2	FTO	activate	NA	IDH2 elevated FTO activity and contributed to tumorigenesis and progression in MM	MM	2021	[177]

*NA* Not reported

## Conclusion and perspective

Despite considerable research underway to understand the function of m^6^A modifications in cancer proliferation and drug resistance, many questions remain unanswered. For example, as a broad RNA modification in eukaryotic messenger RNA, will the m^6^A regulator targeted compounds be a good candidate in tumor therapy? How to focus and target key molecules? How to specifically target the regulatory axis involved in m^6^A to reverse drug resistance in tumor tissue?

The practical significance of m^6^A modifications and regulators heralded a new dawn for targeting m^6^A regulators in therapy. However, few m^6^A-phenotype associated inhibitors and activators are clinically applicable. Followings might be responsible for this plight. Firstly, due to lacking study on cellular activity, how these compounds actually affect methylation levels is elusive. Secondly, adenosine analogs have poor cell permeability and pharmacokinetics, complicating their potential use. Thirdly, tumor heterogeneity and rare predictors mound a barrier between the targeted compounds and distinct cancers, contributing to poor clinical applicability. Therefore, further screening of potential agents is needed. For the precise regulation of m^6^A modifications (global and/or targeted), protein-protein interactions (PPI) or protein-nucleotide interactions would be promising strategies. Further studies on tumor biology, the development of high-quality chemical probes, and preclinical studies will help to identify precise biomarkers, which are crucial for individualized treatment, improved outcomes, and potential toxicity prediction. In addition, most of the reported targeted compounds are cytotoxic, whereas non-cytotoxic inhibitors that modulate the immune system also represent a promising combination. For example, the ALKBH5 inhibitor ALK-04 showed significant synergy with anti-PD-1 therapy while without cytotoxicity *in vivo*. Overall, the clinical application of compounds targeting m^6^A is still in its infancy. As the understanding of epigenomics in cancer grows, there is great promise for those therapy-resistant patients accompanied with abnormal m^6^A manners.

## Data Availability

Not applicable.

## Abbreviations

5-FU: 5-fluorouracil
ABC: ATP-binding cassette
ADR: Adriamycin resistance
ALKBH5: alkb homolog 5
AML: Acute myeloid leukemia
ATG: Associated gene
BC: Breast cancer
BCL-2: B-cell lymphoma 2
BLCA: Bladder cancer
BRCA2: Breast-cancer susceptibility gene 2
CAFs: Cancer-associated fibroblasts
ccRCC: Clear cell renal cell carcinoma
CES2: Carboxylesterase 2
circRNA: Circular RNA
CRC: Colorectal cancer
CSCC: Cervical squamous cell carcinoma
CSCs: Cancer stem cells
CYP2C8: Cytochrome P450 2C8
EGFR: Epidermal growth factor receptor
EMT: Epithelial-to-mesenchymal transition
EOC: Epithelial ovarian cancer
ERRγ: Estrogen-related receptor gamma
ESCC: Esophageal squamous cell carcinoma
FTO: Fat mass and obesity-associated protein
GBM: Glioblastoma multiforme
GC: Gastric cancer
GSCs: Glioblastoma stem cells
HCC: Hepatocellular carcinoma
HNRNP: Heterogeneous nuclear ribonucleoprotein
ICC: Intrahepatic cholangiocarcinoma
IGF2: Insulin-like growth factor 2
IFN-γ: Interferon-gamma
LC3B: Light chain 3B
lncRNA: Long-noncoding RNA
LUAD: Lung adenocarcinoma
m^6^A: N6-methyladenosine
m^6^A_m_: Demethylate N6,2′-O-dimethyladenosine
MA: Meclofenamic acid
MDR: Multidrug resistance
METTL: Methyltransferase-like
miRNA: microRNA
MM: Multiple myeloma
mRNA: Messenger RNA
NKAP: NF-κB activating protein
NKTCL: Natural killer/T-cell lymphoma
NSCLC: Non–small cell lung cancer
OSCC: Oral squamous cell carcinoma
PC: Pancreatic cancer
PD-1: Programmed cell death protein 1
PDK1: Phosphoinositide-dependent kinase-1
PPI: Protein-protein interactions
R-2HG: R-2-hydroxyglutarate
SAM: S-adenosyl-L-methionine
SKCM: Skin cutaneous melanoma
STAT3: Signal transducers and activators of transcription 3
T-ALL: T-cell acute lymphoblastic leukemia
TIME: Tumor immune microenvironment
TKI: Tyrosine kinase inhibitor
UGT: UDP-glucuronosyltransferase
ULK1: Unc-51-like kinase 1
USP: Ubiquitin-specific protease
UTR: Untranslated regions
WTAP: Wilms tumor 1-associated protein
YAP: Yes-associated protein
YTH: YT521-B homology
